# UNDERSTANDING AND ADDRESSING COMMUNITY-BASED PALLIATIVE CARE REFUSALS: A MULTI-PERSPECTIVE STUDY

**DOI:** 10.1093/geroni/igae098.4269

**Published:** 2024-12-31

**Authors:** Kira Sheldon, Elizabeth Luth

**Affiliations:** Rutgers University--New Brunswick, New Brunswick, New Jersey, United States; Rutgers University, New Brunswick, New Jersey, United States

## Abstract

More than half of older adults have multimorbid chronic conditions, resulting in disability, functional limitations, and reduced quality of life. Community-based palliative care, increasingly part of Medicare Advantage value-based care plans, supports seriously ill individuals and their caregivers to improve symptom management and satisfaction and reduce acute care use. Yet, many potentially eligible individuals decline free services when offered. To understand why eligible individuals refuse palliative care, we conducted semi-structured interviews and focus groups with 22 Medicare Advantage plan members and caregivers who refused palliative care and five palliative care team members at a non-profit healthcare agency. Transcripts were analyzed to identify reasons for refusal. We identified three overarching reasons plan members and caregivers declined palliative care: (mis)associating it with end of life, unclear benefit of palliative care, and not wanting to change current care. Palliative care team members confirmed these findings and contributed additional insights. Medicare Advantage plan members and families who refused palliative care represent a key resource for understanding how to better explain services and increase acceptance of palliative care. Palliative care team members’ perspectives provide insights from interactions with seriously ill individuals who may be disinclined or unable to engage in research. In clinical practice, clearly differentiating palliative care from hospice, providing an individualized explanation for how it will address an individual’s particular needs, and emphasizing integration of palliative care with existing care teams may improve engagement in palliative care among seriously ill older adults.

